# Supplementary figures and images for: The effect of pulsed electromagnetic field exposure on osteoinduction of human mesenchymal stem cells cultured on nano-TiO2 surfaces
Source: PLoS One. 2018 Jun 14;13(6):e0199046. doi: 10.1371/journal.pone.0199046 (PMC6002089; doi:10.1371/journal.pone.0199046)

S1 Fig.

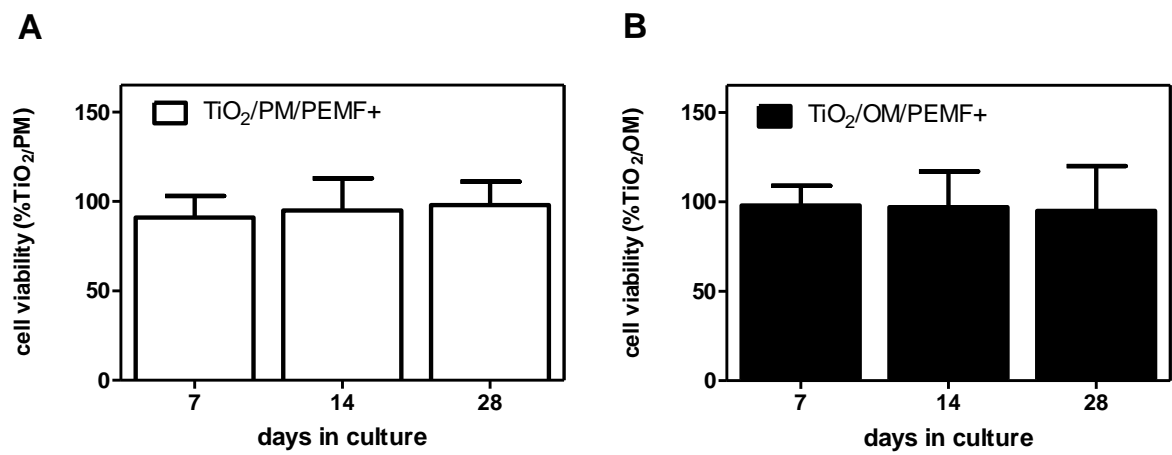

Supplement: S1 Fig — Stem cells grown in proliferative (A) or osteogenic (B) medium and normalized to the unstimulated samples (TiO2-seeded cells) cultured in the same culture medium (PM, A; OM, B). MTT cell viability assay was performed daily at different time frames of culture stimulating stem cells with PEMF. The control was represented by unstimulated culture (TiO2-seeded cells). Data are presented as viability percentage to unstimulated culture set equal to 100%. Bars indicate mean values ± standard error of the mean of results from three experiments. (PDF) [file pone.0199046.s001.pdf]
