# Supplementary material for: The effect of pulsed electromagnetic field exposure on osteoinduction of human mesenchymal stem cells cultured on nano-TiO2 surfaces
Source: PLoS One. 2018 Jun 14;13(6):e0199046. doi: 10.1371/journal.pone.0199046 (PMC6002089; doi:10.1371/journal.pone.0199046)
Supplement: S1 Table — (PDF) [file pone.0199046.s002.pdf]

S1 Table.

Genes	Upstream primer forward 5'- 3'	Downstream primer reverse 5'- 3'	Amplicon size (bp)
**ALP** [a)]	CTA TCC TGG CTC CGT GTC C	AGC CCA GAG ATG CAA TCG	138
**BMP-2** [b)]	CCT CCG TGG GGA TAG AAC TT	CAC TGT GCG CAG CTT CC	107
**BOSP** [c)]	GGG CAG TAG TGA CTC ATC CG	TCA GCC TCA GAG TCT TCA TCT TC	90
**COL-I** [d)]	CAT GTT CAG CTT TGT GGA CC	TTC TGT ACG CAG GTG ATT GG	128
**FN** [e)]	ACC TCG GTG TTG TAA GGT GG	CCA TAA AGG GCA ACC AAG AG	91
**GAPDH** [f)]	AGC CTC AAG ATC ATC AGC AAT GCC	TGT GGT CAT GAG TCC TTC CAC GAT	120
**RUNX-2** [g)]	ACA GTA GAT GGA CCT CGG GA	ATA CTG GGA TGA GGA ATG CG	113
**OSC** [h)]	AAG AGA CCC AGG CGC TAC CT	AAC TCG TCA CAG TCC GGA TTG	107
**OSX** [i)]	CTC AGC TCT CTC CAT CTG CC	GGG ACT GGA GCC ATA GTG AA	99

[a)]ALP, alkaline phosphatase; [b)]BMP-2, bone morphogenetic protein 2; [c)]BOSP, bone sialoprotein; [d)]COL-I, type-I collagen; [e)]FN, fibronectin; [f)]GAPDH, glyceraldehyde-3-phosphate dehydrogenase; [g)]RUNX-2, runt-related transcription factor 2; [h)]OSC, osteocalcin; [i)]OSX, osterix. GAPDH was the housekeeping gene.
