# Supplementary material for: The effect of pulsed electromagnetic field exposure on osteoinduction of human mesenchymal stem cells cultured on nano-TiO2 surfaces
Source: PLoS One. 2018 Jun 14;13(6):e0199046. doi: 10.1371/journal.pone.0199046 (PMC6002089; doi:10.1371/journal.pone.0199046)
Supplement: S2 Table — (PDF) [file pone.0199046.s003.pdf]

**S2 Table.**

| Proteins | Proliferative medium | | |
|---|---|---|---|
| | [a)] $TiO_2$ | [b)] $TiO_2$/PEMF+ | Ratio a/b |
| **ALP** | 6.2 ± 0,68 | 6.8 ± 0,5 | 1.1 |
| **COL-I** | 35 ± 3.00 | 40 ± 2.70 | 1.1 |
| **COL-III** | 36.7 ± 11 | 39.5 ± 9 | 1.0 |
| **DCN** | 40.1 ± 2.4 | 43.0 ± 1.6 | 1.0 |
| **FN** | 8.08 ± 1.01 | 9.00 ± 2.00 | 1.1 |
| **OPN** | 20.07 ± 4,03 | 21.06 ± 2.07 | 1.1 |
| **OSC** | 1.4 ± 0.4 | 1.7 ± 0.6 | 1.2 |
| **OSN** | 1.74 ± 0.33 | 1.93 ± 0.55 | 1.1 |
